# lncRNA OGFRP1 promotes tumor progression by activating the AKT/mTOR pathway in human gastric cancer

**DOI:** 10.18632/aging.202731

**Published:** 2021-03-19

**Authors:** Jingzhou Zhang, Xiujuan Xu, Junfeng Yin, Jiaqi Tang, Nan Hu, Yidong Hong, Ziyan Song, Baoxiang Bian, Fenglei Wu

**Affiliations:** 1 Department of Oncology, Lianyungang Clinical College of Nanjing Medical University, The First People’s Hospital of Lianyungang, Lianyungang, China; 2 Department of Radiation Oncology, Lianyungang Second People's Hospital, Lianyungang, China; 3 Department of General Surgery, The Affiliated Hospital of Yangzhou University, Yangzhou University, Yangzhou, China; 4 Deparment of Oncology, School of Medicine, Jiangsu University, Zhenjiang, Jiangsu, China

**Keywords:** lncRNA, OGFRP1, gastric cancer, proliferation, apoptosis

## Abstract

As biomolecules of great clinical value, lncRNAs play a crucial role as regulators in the processes of tumor origin, metastasis, and recurrence. Thus, lncRNAs are urgently needed for research in gastric cancer. We elucidated the specific function of OGFRP1, both *in vitro* and *in vivo*. OGFRP1 was expressed at abnormally high levels in gastric cancer samples (n = 408) compared to normal samples (n = 211). Similar results were obtained in 30 clinical case samples. Interference of OGFRP1 markedly blocked cell proliferation and migration, and it induced cell cycle arrest and the apoptosis of gastric cancer cells *in vitro*. Phosphorylation of AKT was inhibited in cells transfected with OGFRP1 siRNA, as compared to their control cells. The *in vivo* results further confirmed the antitumor effects of OGFRP1 knockdown on gastric cancer. Decreases in tumor volume (104.23±62.27 mm^3^) and weight (0.1006±0.0488 g) in nude mice were observed during the OGFRP1 interference, as compared with the control group (418.96±211.96 mm^3^ and 0.2741±0.0769 g). OGFRP1 promotes tumor progression through activating the AKT/mTOR pathway. Our findings provide a new potential target for the clinical treatment of human gastric cancer.

## INTRODUCTION

Gastric cancer is the fifth most common malignant tumor in the world. In 2012, approximately 951,600 new cases were diagnosed [1]. Despite the decline in gastric cancer mortality in recent years, it is still the third leading cause of cancer death [2, 3]. At present, tumor markers for early diagnosis of gastric cancer are not ideal due to their low sensitivity and specificity [4, 5]. The treatment of gastric cancer is still based on surgical resection and chemotherapy, but these treatments pose issues such as side effects, recurrence, and metastasis [6]. Although the molecular mechanism research of gastric cancer has made significant progress, there are still many limitations in the application of research results in clinical treatment. Additionally, the mortality rate of gastric cancer is still high. Therefore, elucidating new mechanisms associated with the pathogenesis of gastric cancer is critical for identifying useful biomarkers and developing effective targeted therapies to improve clinical outcomes.

Long non-coding RNAs are RNAs that do not encode a protein with the length of ≥ 200 nucleotides. Numerous studies have confirmed that lncRNAs are widely involved in various biological processes in organisms [7, 8]. In tumors, lncRNAs play a crucial role as important regulators in the processes of tumor origin, metastasis, and recurrence [8]. Although the roles of lncRNAs are extensive, the current research on lncRNAs is extremely limited. Studies have shown that the number of lncRNAs in humans has reached approximately 59,000, only 10,000 of which have been annotated [9]. Moreover, the expression of lncRNAs is characterized by low abundance and high tissue specificity, which makes them generally more difficult to research than protein-coding genes [10]. LncRNAs, as biomolecules of great clinical value, are urgently needed for research and will definitely open up new areas for the treatment of tumors.

In recent years, lncRNA OGFRP1 has been gradually found to be involved in the progression of a variety of tumors, including non-small cell lung cancer [11], cervical carcinoma [12], endometrial cancer [13] and hepatocellular carcinoma [14], revealing its important role in the regulation of tumor cell behavior However, its role in gastric cancer still remains unclear. In the present study, we investigated the specific role of lncRNA OGFRP1 on gastric cancer *in vitro* and *in vivo*.

## RESULTS

### lncRNA OGFRP1was up-regulated in human gastric cancer

**Figure 1:**
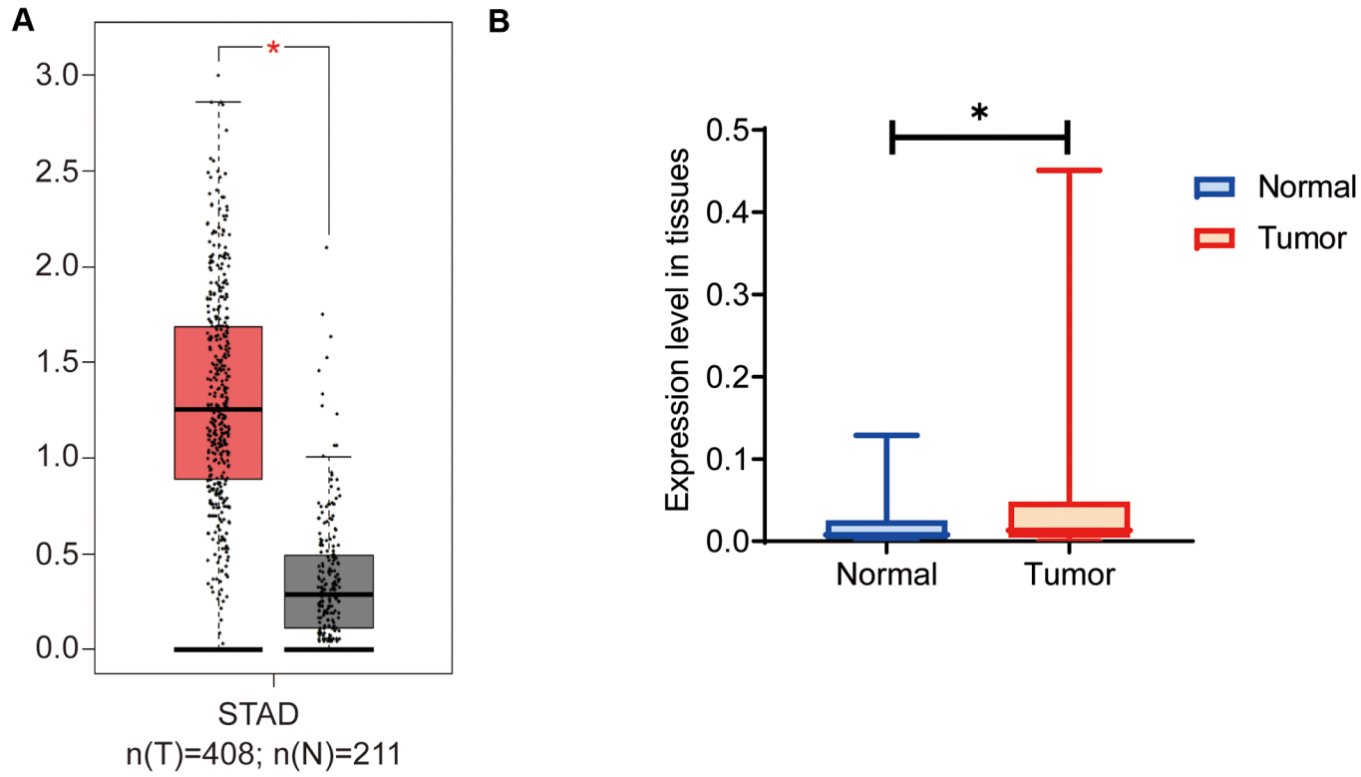
**OGFRP1 expression increased in human gastric cancer tissues.** (**A**) There are 408 cases of gastric cancer and 211 cases of normal gastric tissues included in the GEPIA dataset. The online analysis of OGFRP1 expression was performed, and the results showed that the expression of OGFRP1 was upregulated in gastric cancer (red box) compared with normal gastric cases (gray box). (**B**) The qPCR was performed to analyze OGFRP1 expression in tumor and normal tissues in 30 cases of gastric cancer. *P<0.05.

On GEPIA we analyzed the expression levels of OGFRP1 in gastric cancer. As shown in Figure 1A, the expression of OGFRP1 significantly increased in tumor tissues (red box), as compared to the normal tissues (gray box). To verify the results of the bioinformatics analysis, we further detected OGFRP1 expression in 30 cases of gastric cancer. In comparison to the paired normal tissues, OGFRP1 level increased significantly in gastric cancer (P<0.05, Figure 1B). These observations indicated that lncRNA OGFRP1 was unregulated in gastric cancer.

### The knockdown of OGFRP1 inhibited the proliferation in human gastric cancer cells

**Figure 2:**
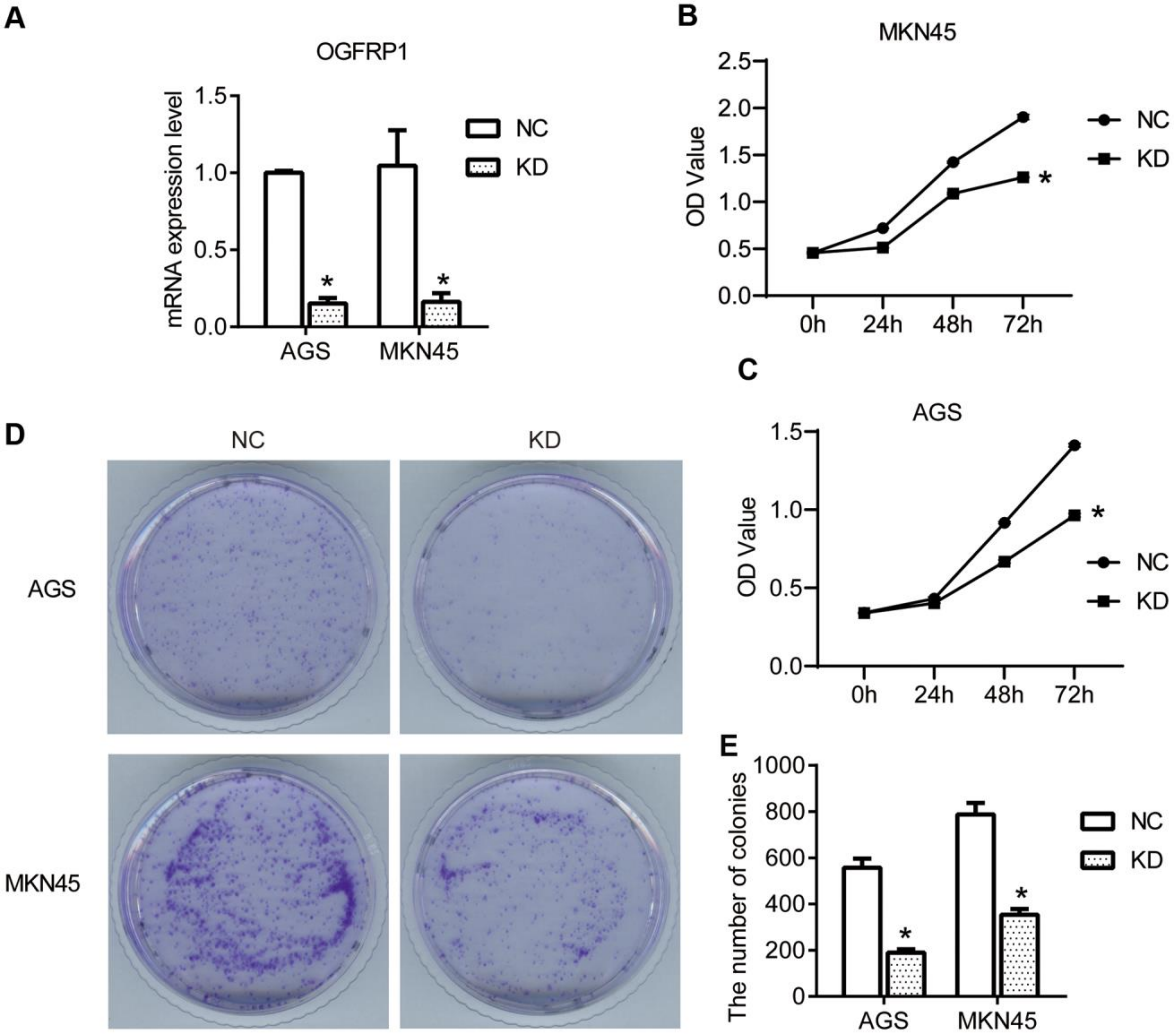
**The knockdown of OGFRP1 inhibited the proliferation in human gastric cancer cells.** (**A**) The expression of OGFRP1 in cells transfected with OGFRP1 specific-siRNA (KD) or control siRNA (NC) was determined by qPCR. CCK8 assay was performed to detect the proliferation of the NC and KD groups in MKN45 (**B**) and AGS cells (**C**). OD values were analyzed by a microplate reader at 450nm. (**D**) The proliferations of MKN45 and AGS cells were further evaluated by clone formation assay. (**E**) Statistical analysis of colony numbers was performed using the *t-test*. *P<0.05.

To confirm our hypothesis, we knocked down the expression of OGFRP1 in two gastric cancer cell lines, AGS and MKN45. We used the transfection of OGFRP1 specific-siRNA (KD), with a control siRNA as the negative control (NC). First, we evaluated the proliferation of the NC and KD groups using CCK8 assay. The results in Figure 2B, 2C demonstrate that the OD value of AGS or MKN45 cells after transfection with OGFRP1 siRNA for 48h and 72h declined significantly, as compared to that of the NC cells (*P*<0.05). This data suggests that OGFRP1 knockdown inhibited the proliferation of gastric cancer cells. The proliferations of MKN45 and AGS cells were further evaluated by clone formation assay. Consistently, colony numbers of OGFRP1 knockdown cells significantly decreased in comparison to that of NC cells (*P*<0.05, Figure 2D, 2E).

### OGFRP1 knockdown inhibited migration by reversing the epithelial-mesenchymal transition (EMT) process in human gastric cancer cells

**Figure 3:**
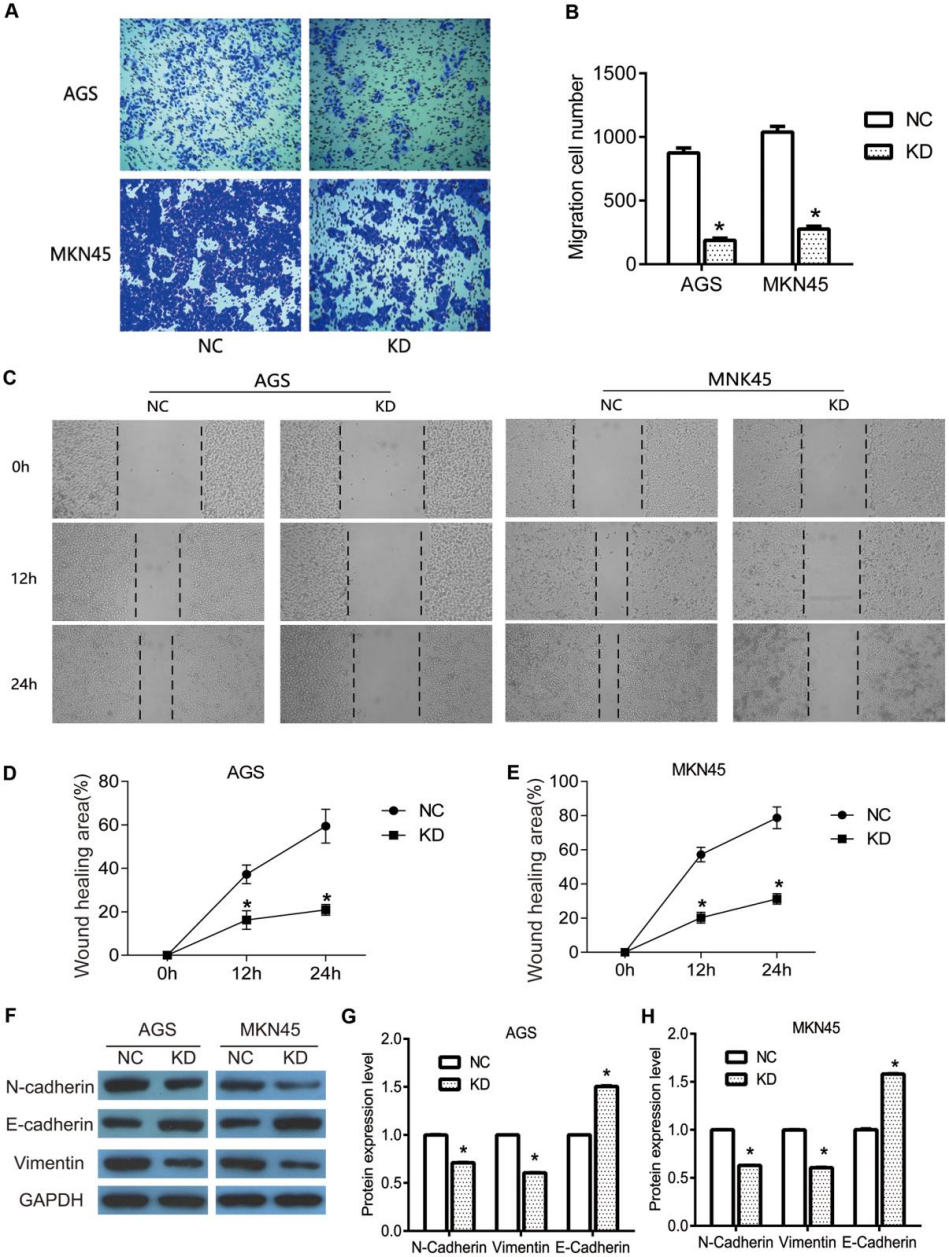
**The knockdown of OGFRP1 inhibited the migration in human gastric cancer cells.** (**A**) The migrations of AGS and MKN45 cells with the transfection of control siRNA or OGFRP1 specific-siRNA were evaluated by transwell. (**B**) Statistical analysis of migration cell number was performed using the *t-test*. (**C**) Wound healing assay was performed to further confirm the migration of AGS and MKN45 cells. The healing speed of scratches was calculated by healing area after scratching for 48 h/ total scratch area. (**D**) Statistical analysis of AGS cells was performed using the *t-test*. (**E**) Statistical analysis of MKN45 cells was performed using the *t-test*. (**F**) The expressions of migration-related proteins were determined using western blot. Statistical analyses of protein expression levels in AGS (**G**) and MKN45 (**H**) cells were performed using the t-test. *P<0.05.

To further investigate the specific role of OGFRP1 in human gastric cancer, transwell and wound healing assays were performed to determine the migration of AGS and MKN45 cells. As shown in Figure 3A, 3B, transwell assay results revealed that the migration cell number of the KD group (AGS, 187±18; MKN45, 276±23) significantly declined in comparison to the NC group (AGS, 875±38; MKN45, 1038±45) (*P*<0.05). Consistent with the results of the transwell assays, the wound healing area of the KD group significantly decreased at 12h and 24h after scratching (*P*<0.05, Figure 3C–3E). This proved that the suppression of OGFRP1 inhibited the migration of gastric cancer cells.

EMT is a potentially embryonic process that can be abnormally activated during the progression of tumors, including in gastric cancer [15]. Because of its ability to provide invasiveness and mobility for cells, it is often considered a major driver of metastasis and transmission. In the present study, we detected the expression of the EMT markers N-cadherin, E-cadherin, and Vimentin. We used western blot to investigate the pathway by which OGFRP1 knockdown inhibited cell migration in gastric cancer. As shown in Figure 3F–3G, OGFRP1 knockdown markedly inhibited the expression of N-cadherin and Vimentin, while it promoted E-cadherin expression in AGS and MKN45 cells (*P*<0.05), as compared to the control group. Taken together, our data suggested that the suppression of OGFRP1 inhibited the migration of gastric cancer cells by reversing the EMT process.

### OGFRP1 knockdown blocked the cell cycle in human gastric cancer cells

**Figure 4:**
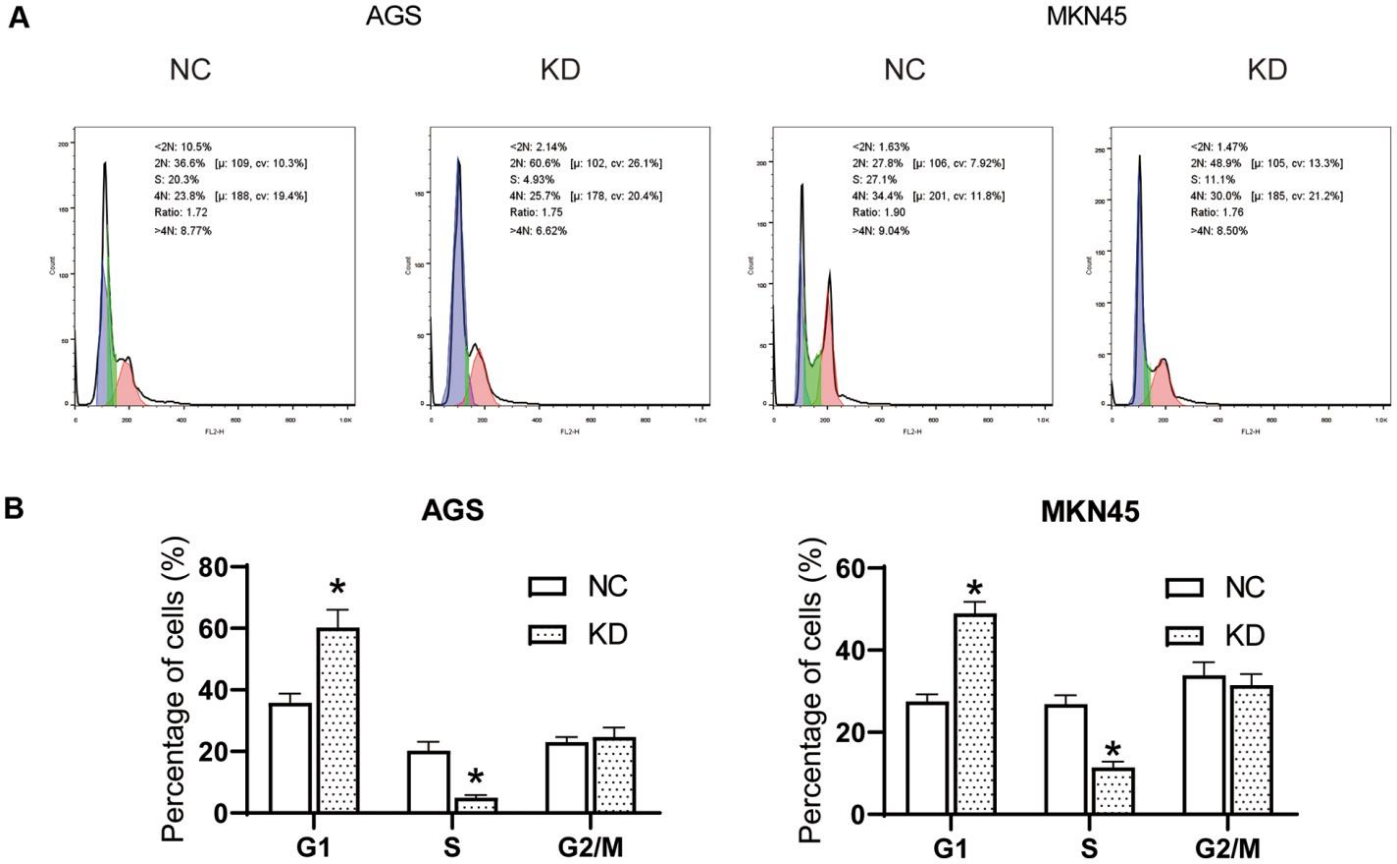
**The knockdown of OGFRP1 blocked the cell cycle in human gastric cancer cells.** (**A**) Flow Cytometry Analysis was performed to analyze the cell cycles of AGS and MKN45 cells. (**B**) The percentage of AGS and MKN45 cells in the G1, S, and G2/M phases were summarized in the bar charts. *P<0.05.

Flow Cytometry Analysis was performed to analyze the cell cycles of AGS and MKN45 cells (Figure 4A). After interfering with OGFRP1 siRNA, the levels of G1 phase in AGS and MKN45 cells increased significantly in comparison to the NC group. Meanwhile, the level of S phase in AGS and MKN45 cells decreased markedly in comparison to the NC group (*P*<0.05, Figure 4B). These observations indicated that after the transfection of OGFRP1 siRNA, the cell cycle was blocked at the G1 phase in AGS MKN45 cells.

### The knockdown of OGFRP1 induced the apoptosis by regulating related proteins in human gastric cancer cells

**Figure 5:**
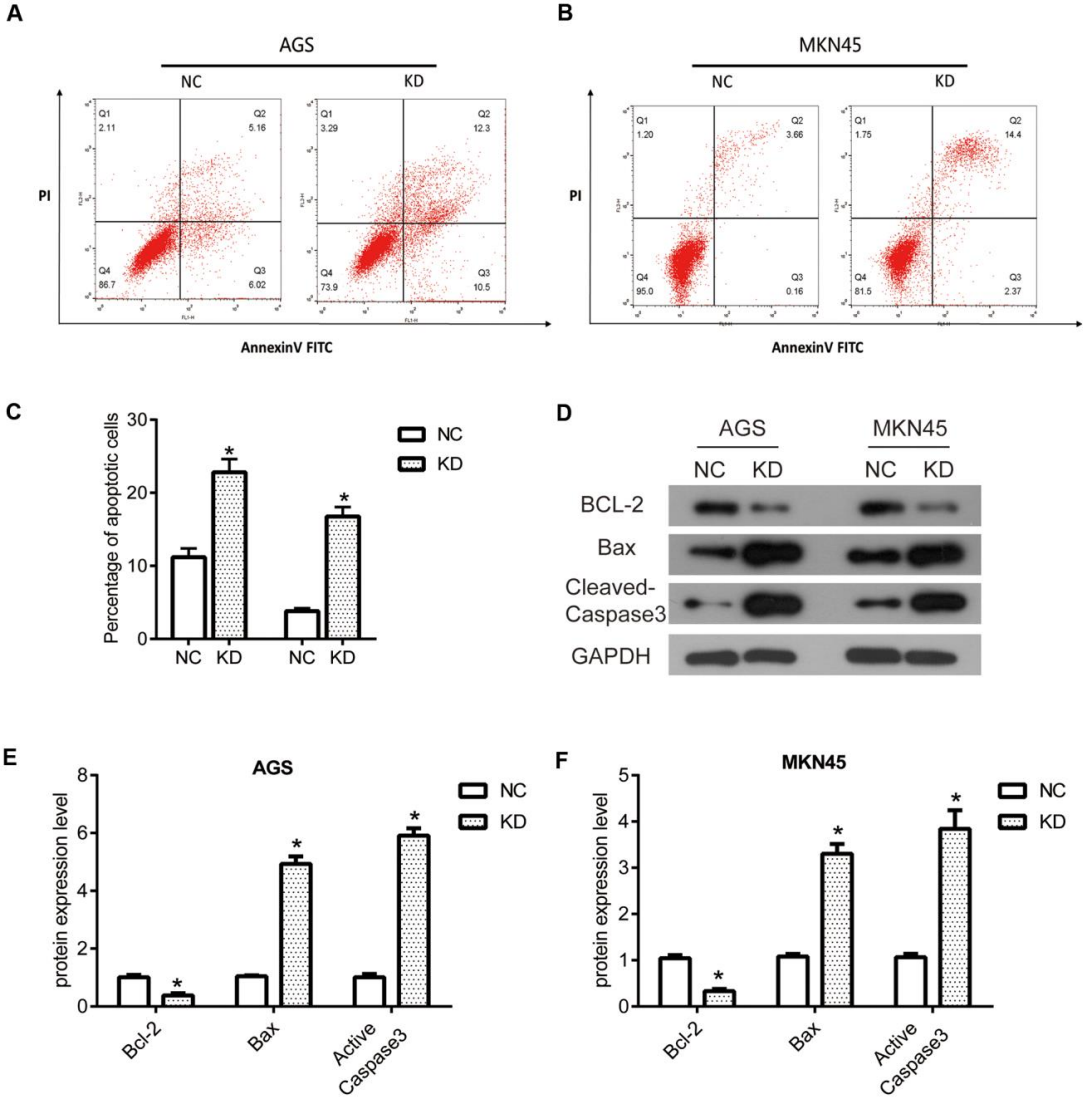
**The knockdown of OGFRP1 induced the apoptosis by regulating related proteins in human gastric cancer cells.** Flow cytometry analysis was performed to evaluate the apoptosis of AGS (**A**) and MKN45 (**B**) cells. (**C**) Statistical analysis of the percentage of apoptosis cells was performed using the *t-test*. (**D**) The expressions of apoptosis-related proteins were determined using western blot. Statistical analysis of protein expression levels in AGS (**E**) and MKN45 (**F**) cells was performed using the *t-test*. *P<0.05.

Flow cytometry analysis was performed to evaluate the apoptosis of AGS and MKN45 cells. As shown in Figure 5A, 5C, the percentage of apoptosis cells increased in AGS cells with the transfection of OGFRP1, as compared to the NC cells. A similar result was also observed in MKN45 cells. As shown in Figure 5B, 5C, the percentage of apoptosis cells significantly increased after transfection with OGFRP1 siRNA for 48h, in comparison to the NC cells. Our data demonstrated that suppression of OGFRP1 induced the apoptosis in human gastric cancer cells. Then, we further explored the molecular mechanism of OGFRP1 affecting apoptosis. The expressions of apoptosis-related proteins were determined using western blot (Figure 5D). From the results showed in Figure 5E, 5F, the levels of apoptosis factor, Cleaved-Caspase3 and Bax, were markedly up-regulated after the suppression of OGFRP1. To the contrary, the expression of the apoptosis inhibitory factor Bcl-2 was down-regulated in OGFRP1 knockdown cells. Taken together, these observations demonstrated that the suppression of OGFRP1 regulated the levels of multiple apoptosis-related factors and induced the apoptosis in gastric cancer cells.

### OGFRP1 knockdown inhibited tumor progression by suppressing the AKT/mTOR pathway in human gastric cancer cells

**Figure 6:**
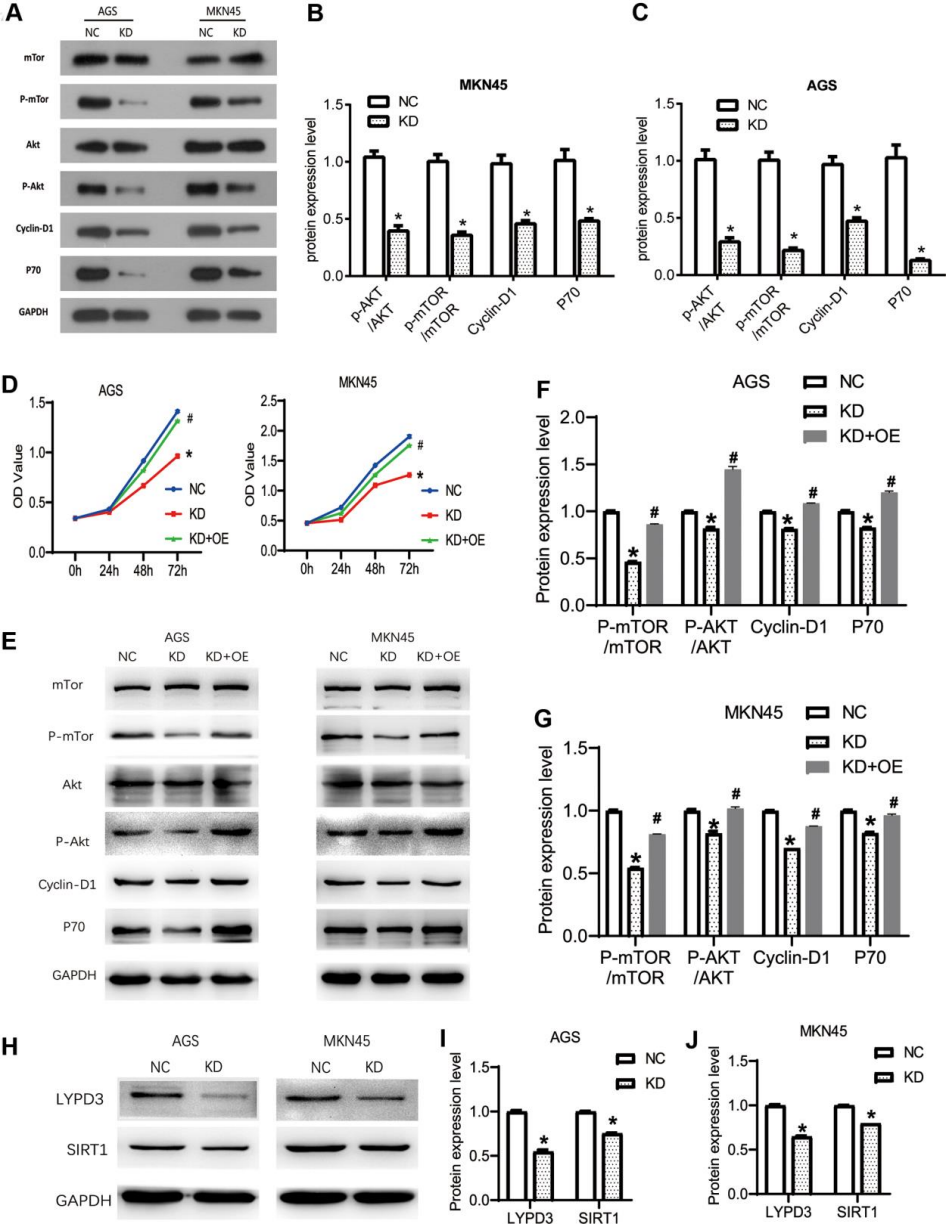
**The knockdown of OGFRP1 inhibited tumor progression by activating the AKT/mTOR pathway in human gastric cancer cells.** (**A**) The activities of AKT/mTOR pathway-related proteins were analyzed using western blot. Statistical analysis of protein expression levels in MKN45 (**B**) and AGS (**C**) cells were performed using the *t-test*. (**D**) OGFRP1 overexpression plasmid was transfected into OGFRP1 knockdown cells (KD+OE group). CCK8 was performed to detect the proliferation of MKN45 and AGS cells. (**E**) The levels of AKT/mTOR pathway-related proteins were detected by western blot in AGS (**F**) and MKN45 (**G**) cells. (**H**) The expression levels of LYPD3 and SIRT1 were detected by western blot in AGS (**I**) and MKN45 (**J**) cells. *P<0.05.

Studies have shown that the AKT pathway can be significantly activated in gastric cancer, acting as a key signaling pathway that regulates cell proliferation, migration, and apoptosis. At the same time, the latest research proves that OGFRP1 can abnormally activate the AKT pathway in hepatocellular carcinoma. Therefore, we speculate that OGFRP1 may participate in tumor progression by regulating this pathway. We performed the western blot to analyze the activities of AKT/mTOR pathway-related proteins (Figure 6A). As shown in Figure 6B, 6C, the levels of AKT and mTOR phosphorylation were significantly inhibited by the interference of OGFRP1 in both AGS and MKN45 cells. Cyclin D1 is a downstream molecule of the AKT signaling pathway and it is directly involved in the regulation of the cell cycle. Our results showed that the protein expression level of Cyclin D1 decreased after the transfection of OGFRP1 siRNA. Moreover, proliferation-related protein P70 was also down-regulated in OGFRP1 knockdown cells compared to the control cells.

To further confirm the effects of OGFRP1 on the AKT/mTOR pathway, we overexpressed OGFRP1 in KD group cells. Then, we detected the proliferation and AKT/mTOR pathway-related protein using CCK8 and western blot, respectively. As shown in Figure 6D, OGFRP1 overexpression could significantly rescue the inhibition of proliferation induced by OGFRP1 knockdown. Further, the phosphorylation levels of AKT and mTOR were also rescued by OGFRP1 overexpression, in addition to the expression of Cyclin D1 and p70 (Figure 6E–6G). These results indicated that the suppression of OGFRP1 played a specific role on tumor progression by inhibiting the AKT/mTOR pathway in gastric cancer.

### OGFRP1 knockdown inhibited the expression of LYPD3 and SIRT1 in gastric cancer cells

In previous reports, OGFRP1 unregulated the expressions of LYPD3 and SIRT1 by sponging miR-124-3p in NSCLC and endometrial cancer, respectively [13, 16]. Thus, we detected the expression levels of LYPD3 and SIRT1 in gastric cancer cells. As shown in Figure 6H–6J, OGFRP1 knockdown could inhibit the expression of LYPD3 and SIRT1 in both AGS and MKN45 cells.

### OGFRP1 knockdown inhibited AGS tumor growth in nude mice

**Figure 7:**
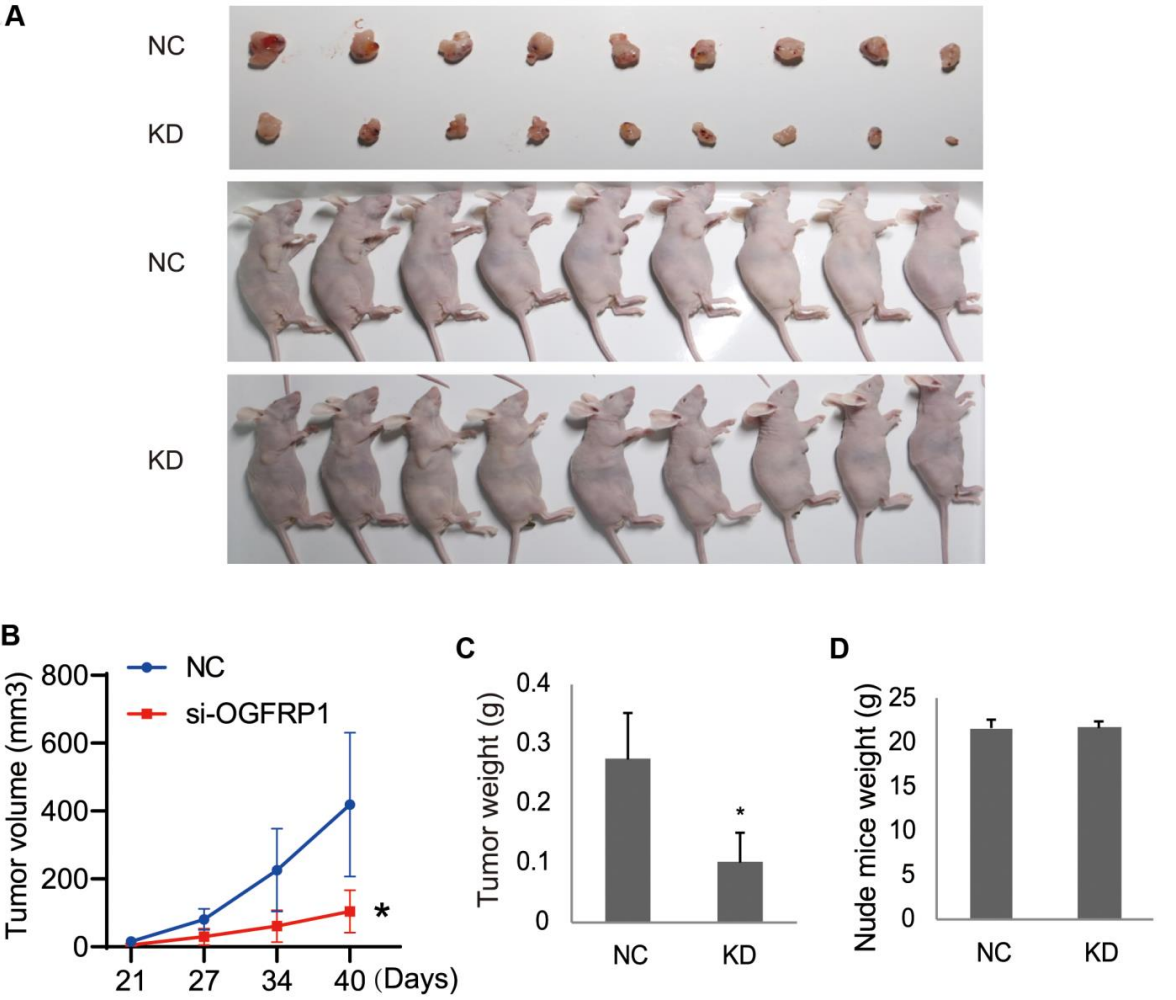
**The knockdown of OGFRP1 inhibited tumor growth in nude mice.** (**A**) Eighteen female BALB/c-nu nude mice were injected in the right flanks with the OGFRP1 knockdown (KD) or the negative control (NC) AGS cells. Surgically removed MDA-MB-231 tumor tissues and nude mice were photographed after 3 weeks of injection. (**B**, **C**) The volumes and weights of the tumors, as well as the weights of the mice (**D**), were recorded and analyzed after 3 weeks of injection. The analysis was performed using the t-test. *P<0.05.

The effects of OGFRP1 knockdown on tumor growth was further investigated *in vivo* by using a nude mice xenograft model. Eighteen female BALB/c-nu nude mice were injected in the right flanks with either OGFRP1 knockdown or the negative control AGS cells (Figure 7A). The volume and weights of the tumors, as well as the weights of the mice, were recorded and analyzed. From our results, the volume of the tumors in the KD group (104.23 ± 62.27 mm^3^) decreased markedly compared with the NC group (418.96 ± 211.96 mm^3^) after 3 weeks of injection (Figure 7C). The weights of the tumors in the KD group (0.1006 ± 0.0488 g) also significantly declined compared with those in the NC group (0.2741 ± 0.0769 g) (Figure 7C). There were no significant changes between the KD (21.7319 ± 0.8085 g) and NC groups (21.6673 ± 1.0332 g) (Figure 7D). These results indicated that OGFRP1 knockdown inhibited tumor growth *in vivo*.

## DISCUSSION

In the present study, we demonstrate the abnormally high expression of lncRNA OGFRP1 in gastric cancer tissues for the first time. We also demonstrate the specific effects of promoting the proliferation and migration of tumor cells, as well as that of inducing cell cycle arrest and apoptosis. Results suggest that OGFRP1 is a potential biomarker and therapeutic target for gastric cancer.

lncRNA, as a kind of non-coding RNA, has not received much attention from researchers. In recent years, it has been reported that lncRNA plays an important role in various diseases [17, 18], and that its significance in tumors is becoming increasingly recognized [19, 20]. Studies have confirmed that lncRNAs can regulate cell function through a variety of mechanisms [21, 22]. lncRNAs can directly affect downstream gene expression as a cis-acting element. They can also affect the expression of a target gene by forming RNA-RNA, RNA-DNA complexes, or degrade mRNA through RNA interference mechanisms [23–26]. Through the above mechanisms, lncRNAs affect the expression and activity of genes, and they can change the biological state of cells, thereby participating in the regulation of tumor origin and progression. At present, a number of functional lncRNAs have been identified. For example, lncRNA MEG3 can inhibit the proliferation of cervical cancer cells, while its expression in clinical cervical cancer specimens is significantly down-regulated, indicating that lncRNA MEGS plays an important role as a tumor suppressor gene [27, 28]. LncRNA MVIH is expressed in hepatic tumor tissue, promotes angiogenesis, and leads to a worse prognosis [29]. The roles of lncRNAs in the development of tumorigenesis are being gradually elucidated, and the research of their roles in gastric cancer has gradually increased. A group of functional lncRNA has also been found in gastric cancer. It has been reported that HOTAIR, ANRIL, GAPLINC, GHET1, H19, GAS5, and FENDRR, etc., play roles in the malignant progression of gastric cancer [30–36].

The aim of this study was to try to discover new lncRNA involved in the progression of gastric cancer. First, we demonstrated that OGFRP1 is highly expressed in gastric cancer by using a bioinformatics analysis. OGFRP1 has been studied in liver cancer, endometrial cancer, cervical carcinoma and NSCLC, where it has been found to promote tumor progression [11–14]. To further confirm the overexpression of OGFRP1, we collected tumor and adjacent tissues of 30 cases with gastric cancer, of which OGFRP1 levels were detected by qPCR. Consistently, OGFRP1 was proven to be up-regulated in gastric cancer tissues, suggesting its specific role in human gastric cancer. Then, we detected the proliferation, migration cell cycle, and apoptosis of OGFRP1 knockdown cells. Our results proved that the suppression of OGFRP1 inhibited the proliferation and migration, and induced cell cycle arrest and apoptosis in gastric cancer cells *in vitro*. Taken together, our results confirmed that OGFRP1 operated as a tumor promotor, and its expression might be posed as a predictor for tumor metastasis of human gastric cancer. To explore more into the mechanisms of OGFRP1 in gastric cancer, we detected the expression and activity in cell groups of key proteins in the key pathway of tumor progression, which is the AKT pathway. Phosphorylations of AKT and mTOR were significantly inhibited in cells transfected with OGFRP1 siRNA, as compared to their control group in AGS and MKN45 cell lines. Thus, we predicted that the OGFRP1 knockdown blocked the proliferation by inhibiting the activity of the AKT signaling pathway in human gastric cancer cells. The *in vivo* results further confirmed the antitumor effects of OGFRP1 knockdown on gastric cancer. Decreases in tumor volume and weight were observed in the OGFRP1 knockdown of nude mice compared with the control group.

In conclusion, we found that lncRNA OGFRP1 was up-regulated in gastric cancer through analysis in both online databases and clinical samples. OGFRP1 knockdown blocks cell proliferation and migration, and it induces cell cycle arrest and apoptosis by inhibiting the activity of the AKT/mTOR pathway in human gastric cancer. Our results contribute to expanding the knowledge of OGFRP1 functions in promoting tumor progression, as well as provide a potential target for the clinical treatment of human gastric cancer.

## MATERIALS AND METHODS

### Samples and cell lines

For the detection of OGFRP1 expression, samples from 30 cases of gastric cancer were collected in the first people’s hospital of Lianyungang from September to December 2017. All patients signed informed consent. The human gastric cancer cell lines AGS and MKN45 were ordered from the Cell Resource Database of the Chinese Academy of Sciences (Shanghai, China). DMEM medium with a supplement of 10% fetal bovine serum (FBS), 100 U/ml penicillin, and 0.1 mg/ml streptomycin was used in this research. When the cell density reached 60%, either the OGFRP1 specific or the control siRNA (50nM, Ruibo, China) was transfected into AGS or MKN45 cells using Lipofectamine2000 (Invitrogen, USA). Total RAN was extracted after 24 h of transfection and protein was extracted after 48 h of transfection. OGFRP1 knockdown lentivirus packaged plasmids (Oligobio, Beijing, China) were used to construct stable OGFRP1 knockdown cell lines. Cells (5×10^4^ cells/well) were inoculated in 24-well plates and then lentivirus packaged plasmids (2ug/ml, MOI=10) were added when the cell fusion degree reached about 30%. Three days after infection, GFP expression of the lentivirus reporter gene was observed. Cells with a fluorescence rate of more than 80% were used in the tumor xenograft assay.

### Bioinformatical analysis

The expression of OGFRP1 in gastric cancer was analyzed by the Gene Expression Profiling Interactive Analysis (GEPIA), which is a cancer-related RNA expression analysis website. The data on the GEPIA comes from TCGA and GTEx databases [37]. There are 408 cases of gastric cancer and 211 cases of normal gastric tissues included in the GEPIA dataset.

### Quantitative real-time PCR

After transfection for 24 h, total RAN was extracted from AGS and MKN45 cells using trizol reagent, and then reverse transcribed to cDNA. The expression of lncOGFRP1 was performed by SYBR Premix Ex Taq II. The relative quantification was identified by the 2^-∆∆Ct^ method after standardization to the GAPDH level. The primers OGFRP1-F, 5’-GGATACTGAGAGTGCACAAA-3’ and OGFRP1-F, 5’-CATAACTTTAGAGCCTCCCC-3’ were used in this research.

### CCK8 assay

About 1×10^3^ cells transfected with siRNA were seeded into each well of a 96-well plate. 10 μl of CCK8 reagent (Solarbio Science and Technology, China) was added to each well to determine the proliferation. After incubation at 37° C for 2 h, the OD value of excitation light was analyzed by using an enzyme standard instrument at 450 nm. The proliferation was determined every 24 hours.

### Clone formation assay

After transfection, cells in the logarithmic growth phase were inoculated into a 35 mm cell culture dish at a density of 200 cells/dish. Then, they were cultured at 37° C for 2 to 3 weeks until cells formed sufficiently large clones. We subsequently added 4% paraformaldehyde and maintained the cells for 20 min. Cells were stained with crystal violet for 30 minutes. We counted the number of clones larger than 10 cells under a microscope. The number of colonies = number of clones / number of cells inoculated.

### Transwell assay

Transwell assay was performed to determine the migration of AGS and MKN45 cells. After the transfection for 24h, cells were trypsinized and re-suspended in serum-free medium. About 1×10^4^ cells were added to the upper chamber, while complete medium was added to the lower chamber. This was followed by incubation for 24h. The migrated cells were fixed with 4% paraformaldehyde, stained with 0.1% crystal violet, and photographed under a microscope.

### Western blotting

After transfection for 48h, total proteins from AGS and MKN45 cells were extracted by RIPA buffer containing protease inhibitors and phosphatase inhibitors. The proteins were separated by SDS-PAGE and transferred to a PVDF membrane. Then, the membrane was preblocked and incubated overnight at 4° C with primary antibodies (1:1000). This was followed by the secondary antibodies (1:5000) at room temperature for 1 h. ECL substrate reagent was used to generate chemiluminescent signals. The relative expression levels of proteins were normalized against GAPDH and quantified with QUANTITY ONE software. Antibodies used in this study were as follows: Rabbit anti-human Bcl-2, Bax, Cleaved-Caspase3, AKT, p-AKT, mTOR, p-mTOR, Cyclin D1, and p70 were all ordered from Abcam (USA); Primary antibody, anti-GAPDH (1:5000), and HRP secondary antibodies (1:5000) were purchased from PTG Company (USA). Image Pro Plus 6.0 software was used to calculate the grey value of bands.

### Flow Cytometry Analysis for cell cycle

After transfection, AGS or MKN45 cells were starved for 24h. Cells (1-5×10^6^/ml) were fixed overnight with 70% ethanol. The fixed cells were measured by 100μl RNase at 37° C for 30min and then stained with 400μl PI (Roche, Switzerland) in a dark environment. Red fluorescence was detected at 488nm.

### Flow cytometry analysis for the apoptosis

The Annexin V-FITC Apoptosis Detection Kit I was used for the analysis of cell apoptosis. After transfection for 48 h, AGS or MKN45 cells were harvested and washed with precooled PBS. Then, cells were centrifuged and the supernatant was carefully removed. Annexin V binding buffer was added to resuspend cells to 1 - 5 × 10^6^ / ml. 100 μl cell suspension was incubated with 5 μl Annexin V / FITC mix for 5 min. Statistical analysis was performed using Flowjo software.

### Tumor xenograft assay

Eighteen female BALB/c-nu nude mice (4-6 weeks old) were purchased from the Beijing Vital River Laboratory Animal Technology Co., Ltd. and randomly divided into two groups. The mice were maintained by the standard procedures. AGS cells were transfected with lentivirus packaged plasmids, which were purchased from Oligobio (Beijing, China). Cells were then collected into suspensions with a concentration of 1×10^7^/ml. 100μl cell suspension was inoculated subcutaneously to the right flank of each nude mouse. The long (a) and short (b) diameters of the tumors were measured every six days, and the volume of the tumors was calculated by V = a×b^2^/2. The weights of the mice were recorded every six days, and the weights of the tumors were recorded 40 days after injection. This study was approved by the Regulation of Local Institutional Animal Care and Use Committee (IACUC).

### Statistical analyses

SPSS 18.0 software was utilized to perform statistical analysis. All experiments were repeated three times. The comparison in each group was performed by using a *t-test*. Data was displayed as mean ± SD. *P*<0.05 was considered statistically significant.

### Ethics approval and consent to participate

All the experiments in this study were approved by the local ethics committee.

### Consent for publication

All the authors agree to the publication clause.

### Data availability statement

The data that support the findings of this study are available from the corresponding author upon reasonable request.

## References

[1] Ferlay J, Soerjomataram I, Dikshit R, Eser S, Mathers C, Rebelo M, Parkin DM, Forman D, Bray F. Cancer incidence and mortality worldwide: sources, methods and major patterns in GLOBOCAN 2012. Int J Cancer. 2015; 136:E359–86. 10.1002/ijc.29210 25220842

[2] Li P, Chen S, Chen H, Mo X, Li T, Shao Y, Xiao B, Guo J. Using circular RNA as a novel type of biomarker in the screening of gastric cancer. Clin Chim Acta. 2015; 444:132–36. 10.1016/j.cca.2015.02.018 25689795

[3] Li T, Mo X, Fu L, Xiao B, Guo J. Molecular mechanisms of long noncoding RNAs on gastric cancer. Oncotarget. 2016; 7:8601–12. 10.18632/oncotarget.6926 26788991 PMC4890990

[4] Cui L, Zhang X, Ye G, Zheng T, Song H, Deng H, Xiao B, Xia T, Yu X, Le Y, Guo J. Gastric juice MicroRNAs as potential biomarkers for the screening of gastric cancer. Cancer. 2013; 119:1618–26. 10.1002/cncr.27903 23335180

[5] Fang XY, Pan HF, Leng RX, Ye DQ. Long noncoding RNAs: novel insights into gastric cancer. Cancer Lett. 2015; 356:357–66. 10.1016/j.canlet.2014.11.005 25444905

[6] Karimi P, Islami F, Anandasabapathy S, Freedman ND, Kamangar F. Gastric cancer: descriptive epidemiology, risk factors, screening, and prevention. Cancer Epidemiol Biomarkers Prev. 2014; 23:700–13. 10.1158/1055-9965.EPI-13-1057 24618998 PMC4019373

[7] Min L, Garbutt C, Tu C, Hornicek F, Duan Z. Potentials of long noncoding RNAs (LncRNAs) in sarcoma: from biomarkers to therapeutic targets. Int J Mol Sci. 2017; 18:731. 10.3390/ijms18040731 28353666 PMC5412317

[8] Evans JR, Feng FY, Chinnaiyan AM. The bright side of dark matter: lncRNAs in cancer. J Clin Invest. 2016; 126:2775–82. 10.1172/JCI84421 27479746 PMC4966302

[9] Rönnau CG, Verhaegh GW, Luna-Velez MV, Schalken JA. Noncoding RNAs as novel biomarkers in prostate cancer. Biomed Res Int. 2014; 2014:591703. 10.1155/2014/591703 25243154 PMC4163346

[10] Bush SJ, Muriuki C, McCulloch ME, Farquhar IL, Clark EL, Hume DA. Cross-species inference of long non-coding RNAs greatly expands the ruminant transcriptome. Genet Sel Evol. 2018; 50:20. 10.1186/s12711-018-0391-0 29690875 PMC5926538

[11] Ding Y, Liu JH. The signature lncRNAs associated with the lung adenocarcinoma patients prognosis. Math Biosci Eng. 2019; 17:1593–603. 10.3934/mbe.2020083 32233597

[12] Zou K, Yu H, Chen X, Ma Q, Hou L. Silencing long noncoding RNA OGFRP1 inhibits the proliferation and migration of cervical carcinoma cells. Cell Biochem Funct. 2019; 37:591–97. 10.1002/cbf.3435 31512281

[13] Lv Y, Chen S, Wu J, Lin R, Zhou L, Chen G, Chen H, Ke Y. Upregulation of long non-coding RNA OGFRP1 facilitates endometrial cancer by regulating miR-124-3p/SIRT1 axis and by activating PI3K/AKT/GSK-3β pathway. Artif Cells Nanomed Biotechnol. 2019; 47:2083–90. 10.1080/21691401.2019.1617727 31131636

[14] Chen W, You J, Zheng Q, Zhu YY. Downregulation of lncRNA OGFRP1 inhibits hepatocellular carcinoma progression by AKT/mTOR and Wnt/β-catenin signaling pathways. Cancer Manag Res. 2018; 10:1817–26. 10.2147/CMAR.S164911 29997441 PMC6033083

[15] Zhang H, Liu L, Wang Y, Zhao G, Xie R, Liu C, Xiao X, Wu K, Nie Y, Zhang H, Fan D. KLF8 involves in TGF-beta-induced EMT and promotes invasion and migration in gastric cancer cells. J Cancer Res Clin Oncol. 2013; 139:1033–42. 10.1007/s00432-012-1363-3 23504025 PMC11824695

[16] Tang LX, Chen GH, Li H, He P, Zhang Y, Xu XW. Long non-coding RNA OGFRP1 regulates LYPD3 expression by sponging miR-124-3p and promotes non-small cell lung cancer progression. Biochem Biophys Res Commun. 2018; 505:578–85. 10.1016/j.bbrc.2018.09.146 30274775

[17] Maass PG, Rump A, Schulz H, Stricker S, Schulze L, Platzer K, Aydin A, Tinschert S, Goldring MB, Luft FC, Bähring S. A misplaced lncRNA causes brachydactyly in humans. J Clin Invest. 2012; 122:3990–4002. 10.1172/JCI65508 23093776 PMC3485082

[18] Chen G, Wang Z, Wang D, Qiu C, Liu M, Chen X, Zhang Q, Yan G, Cui Q. LncRNADisease: a database for long-non-coding RNA-associated diseases. Nucleic Acids Res. 2013; 41:D983–86. 10.1093/nar/gks1099 23175614 PMC3531173

[19] Yang G, Lu X, Yuan L. LncRNA: a link between RNA and cancer. Biochim Biophys Acta. 2014; 1839:1097–109. 10.1016/j.bbagrm.2014.08.012 25159663

[20] Peng WX, Koirala P, Mo YY. LncRNA-mediated regulation of cell signaling in cancer. Oncogene. 2017; 36:5661–67. 10.1038/onc.2017.184 28604750 PMC6450570

[21] Xue X, Yang YA, Zhang A, Fong KW, Kim J, Song B, Li S, Zhao JC, Yu J. LncRNA HOTAIR enhances ER signaling and confers tamoxifen resistance in breast cancer. Oncogene. 2016; 35:2746–55. 10.1038/onc.2015.340 26364613 PMC4791209

[22] Han P, Li JW, Zhang BM, Lv JC, Li YM, Gu XY, Yu ZW, Jia YH, Bai XF, Li L, Liu YL, Cui BB. The lncRNA CRNDE promotes colorectal cancer cell proliferation and chemoresistance via miR-181a-5p-mediated regulation of Wnt/β-catenin signaling. Mol Cancer. 2017; 16:9. 10.1186/s12943-017-0583-1 28086904 PMC5237133

[23] Zhang B, Arun G, Mao YS, Lazar Z, Hung G, Bhattacharjee G, Xiao X, Booth CJ, Wu J, Zhang C, Spector DL. The lncRNA Malat1 is dispensable for mouse development but its transcription plays a cis-regulatory role in the adult. Cell Rep. 2012; 2:111–23. 10.1016/j.celrep.2012.06.003 22840402 PMC3408587

[24] Chen S, Wang M, Yang H, Mao L, He Q, Jin H, Ye ZM, Luo XY, Xia YP, Hu B. LncRNA TUG1 sponges microRNA-9 to promote neurons apoptosis by up-regulated Bcl2l11 under ischemia. Biochem Biophys Res Commun. 2017; 485:167–73. 10.1016/j.bbrc.2017.02.043 28202414

[25] Xiong H, Ni Z, He J, Jiang S, Li X, He J, Gong W, Zheng L, Chen S, Li B, Zhang N, Lyu X, Huang G, et al. LncRNA HULC triggers autophagy via stabilizing Sirt1 and attenuates the chemosensitivity of HCC cells. Oncogene. 2017; 36:3528–40. 10.1038/onc.2016.521 28166203

[26] Li C, Wang S, Xing Z, Lin A, Liang K, Song J, Hu Q, Yao J, Chen Z, Park PK, Hawke DH, Zhou J, Zhou Y, et al. A ROR1-HER3-lncRNA signalling axis modulates the hippo-YAP pathway to regulate bone metastasis. Nat Cell Biol. 2017; 19:106–19. 10.1038/ncb3464 28114269 PMC5336186

[27] Zhang J, Yao T, Wang Y, Yu J, Liu Y, Lin Z. Long noncoding RNA MEG3 is downregulated in cervical cancer and affects cell proliferation and apoptosis by regulating miR-21. Cancer Biol Ther. 2016; 17:104–13. 10.1080/15384047.2015.1108496 26574780 PMC4847830

[28] Zhang J, Lin Z, Gao Y, Yao T. Downregulation of long noncoding RNA MEG3 is associated with poor prognosis and promoter hypermethylation in cervical cancer. J Exp Clin Cancer Res. 2017; 36:5. 10.1186/s13046-016-0472-2 28057015 PMC5216566

[29] Cheng S, Wang L, Deng CH, Du SC, Han ZG. ARID1A represses hepatocellular carcinoma cell proliferation and migration through lncRNA MVIH. Biochem Biophys Res Commun. 2017; 491:178–82. 10.1016/j.bbrc.2017.07.072 28716731

[30] Lan WG, Xu DH, Xu C, Ding CL, Ning FL, Zhou YL, Ma LB, Liu CM, Han X. Silencing of long non-coding RNA ANRIL inhibits the development of multidrug resistance in gastric cancer cells. Oncol Rep. 2016; 36:263–70. 10.3892/or.2016.4771 27121324

[31] Pan W, Liu L, Wei J, Ge Y, Zhang J, Chen H, Zhou L, Yuan Q, Zhou C, Yang M. A functional lncRNA HOTAIR genetic variant contributes to gastric cancer susceptibility. Mol Carcinog. 2016; 55:90–96. 10.1002/mc.22261 25640751

[32] Correction: long noncoding RNA GAPLINC regulates CD44-dependent cell invasiveness and associates with poor prognosis of gastric cancer. Cancer Res. 2015; 75:3683. 10.1158/0008-5472.CAN-15-1838 26286476

[33] Huang H, Liao W, Zhu X, Liu H, Cai L. Knockdown of long noncoding RNA GHET1 inhibits cell activation of gastric cancer. Biomed Pharmacother. 2017; 92:562–68. 10.1016/j.biopha.2017.05.088 28577495

[34] Liu G, Xiang T, Wu QF, Wang WX. Long noncoding RNA H19-derived miR-675 enhances proliferation and invasion via RUNX1 in gastric cancer cells. Oncol Res. 2016; 23:99–107. 10.3727/096504015X14496932933575 26931432 PMC7838630

[35] Zhang N, Wang AY, Wang XK, Sun XM, Xue HZ. GAS5 is downregulated in gastric cancer cells by promoter hypermethylation and regulates adriamycin sensitivity. Eur Rev Med Pharmacol Sci. 2016; 20:3199–205. 27466992

[36] Xu TP, Huang MD, Xia R, Liu XX, Sun M, Yin L, Chen WM, Han L, Zhang EB, Kong R, De W, Shu YQ. Decreased expression of the long non-coding RNA FENDRR is associated with poor prognosis in gastric cancer and FENDRR regulates gastric cancer cell metastasis by affecting fibronectin1 expression. J Hematol Oncol. 2014; 7:63. 10.1186/s13045-014-0063-7 25167886 PMC4237812

[37] Tang Z, Li C, Kang B, Gao G, Li C, Zhang Z. GEPIA: a web server for cancer and normal gene expression profiling and interactive analyses. Nucleic Acids Res. 2017; 45:W98–W102. 10.1093/nar/gkx247 28407145 PMC5570223

